# 外周血循环浆细胞流式细胞术检测在浆细胞病中的应用中国专家共识（2024年版）

**DOI:** 10.3760/cma.j.cn121090-20240117-00026

**Published:** 2024-04

**Authors:** 中华医学会血液学分会浆细胞疾病学组 中华医学会血液学分会实验诊断学组

**Keywords:** 浆细胞病, 流式细胞术, 循环浆细胞, Plasma cell disease, Flow cytometry, Circulating plasma cells

## Abstract

流式细胞术检测外周血循环浆细胞（circulating plasma cells，CPCs）在浆细胞病的诊疗中已经展示出了重要价值。为促进流式细胞术检测CPCs在临床中的规范使用，特制定本共识。本共识依据循证原则，明确了意义未明的单克隆免疫球蛋白血症、冒烟型骨髓瘤、多发性骨髓瘤和浆细胞白血病进行CPCs检测的时机与价值，并对CPCs的流式细胞术检测方法进行规范。

## Abstract (en)

Flow cytometry plays an important role in the diagnosis and treatment of plasma cell diseases, particularly in the detection of circulating plasma cells (CPCs) in the peripheral blood. A consensus about the normalized use of flow cytometry in detection of CPCs in peripheral blood in clinical practice has been achieved. This consensus is founded on evidence-based principles, which elucidates the timing and value of flow cytometry for the detection of CPCs in the monoclonal gammopathy of undetermined significance, smoldering myeloma, multiple myeloma, and plasma cell leukemia and standardizes flow cytometry in the detection of CPCs in plasma cell diseases.

浆细胞病是一组克隆性浆细胞增殖性疾病，包括意义未明的单克隆免疫球蛋白血症（MGUS）、多发性骨髓瘤（MM）、浆细胞瘤（孤立性骨浆细胞瘤和髓外浆细胞瘤）、免疫球蛋白沉积病（原发性轻链型淀粉样变、轻链和重链沉积病）、POEMS综合征等。

浆细胞病的早期临床表现具有一定的隐匿性，诊断所需时间较长。传统的骨髓穿刺等检查为有创性，难以满足浆细胞病早期筛查的需求。近年来随着液体活检技术的兴起，相对无创的外周血检测技术应运而生。流式细胞术（FCM）经过20多年的发展，在临床检验中的应用逐渐成熟。大量研究表明，应用FCM检测外周血循环浆细胞（circulating plasma cells，CPCs）有助于浆细胞病的早期筛查、预后判断以及治疗选择[1]–[2]。

本共识以当前外周血CPCs检测在浆细胞病中应用的循证数据为依据进行总结及建议，同时对FCM检测外周血CPCs的技术要点进行推荐，以期规范FCM检测CPCs在临床实践中的应用。文献证据分级依照英国牛津大学医学中心证据分级标准。

一、CPCs在浆细胞病中的临床应用

浆细胞病是一个发生发展的过程，随着疾病发展，恶性浆细胞从骨髓进入外周血，形成外周血CPCs，在肿瘤远处播散中起到重要作用[1]。CPCs可反映肿瘤生物学和肿瘤免疫微环境特征。在浆细胞病治疗前后及随访过程中采用FCM检测CPCs，不仅有助于判断预后、改良现有模型风险分层能力、预测疾病进展、进行疗效评估并指导治疗，还能早期识别出耐药和需要抢先治疗的患者。

1. MGUS：MGUS是一种无症状的癌前病变。MGUS患病率随年龄增长明显增加，美国人群50岁及以上为3.2％，70岁及以上为5.3％，85岁及以上为8.9％[3]。中国北京一项流行病学调查显示MGUS患病率50岁及以上为1.11％，70岁及以上为2.57％[4]。所有的MM均由MGUS进展而来，每年大约1％的MGUS患者进展为MM、淀粉样变性、轻链沉积病等，而接受严密随诊的MGUS患者的总生存率更高、并发症更少[3]。因此，采用灵敏度高、适用性强的方法对MGUS患者进行监测可能具有重要价值。

多项研究显示MGUS患者的外周血CPCs阳性率为21％～59％[5]–[6]（2b）。除目前较为公认的梅奥MGUS风险分层模型外，一项来自欧洲的研究显示CPCs数量高（≥0.058个/µl）的MGUS患者进展至冒烟型骨髓瘤（smoldering multiple myeloma，SMM）或活动性MM的比例显著高于CPCs低（<0.058个/µl）的患者（21％对0，*P*<0.0001）[5]（2b）。提示CPCs可预测MGUS进展风险，未来或可纳入MGUS危险分层标准。

共识1：为准确了解MGUS疾病状况并预判疾病进展风险，患者诊断时和随访过程中，可以在常规检测项目基础上通过FCM检测CPCs。推荐强度：建议采用。

2. SMM：SMM是MM的前期状态，40岁及以上人群的SMM患病率可达0.5％[7]。SMM诊断后的前5年内，每年大约10％的患者进展为MM[8]。目前较常用的SMM风险评估模型包括梅奥模型和西班牙模型，但是两个模型一致性欠佳，临床如何识别出高危SMM仍具有挑战。研究显示CPCs水平升高是SMM进展的独立预后不良因素，能明显提高识别高危SMM患者的能力，可以指导临床进行更加密切随访。

梅奥的一项研究显示，梅奥风险模型［高危组：骨髓浆细胞≥10％，血清单克隆免疫球蛋白（M蛋白）≥3 g/dl］预测SMM患者2年内进展为MM的风险特异性为85％，但阳性预测值（PPV）仅有33％。而以CPCs≥0.1％（即每150 000个外周血细胞中检出150个异常克隆浆细胞）为临界值进行预测，特异性高达97％，PPV为78％，CPCs的预测效能明显优于梅奥模型[9]（2b）。

梅奥20/2/20预测模型［血清游离轻链比值（sFLCR）>20，M蛋白>2 g/dl，骨髓浆细胞>20％］已广泛应用于临床，但在iMMunocell多中心研究中该模型并未得到证实。同时该研究表明CPCs>0.015％（相当于外周血检出0.773个异常克隆浆细胞/µl）是独立的预后不良因素。因此提出了包含CPCs的新SMM模型20/2/0.015（sFLCR>20，M蛋白>2 g/dl，CPCs>0.015％），将患者分为低、中、高三个危险组，低、中危组的疾病进展时间（time to progression，TTP）尚未达到，而高危组患者的TTP为21.3个月。因此，FCM检测CPCs可能有助于SMM的危险分层，完善了20/2/20模型[10]（2b）。FCM检测CPCs应用于SMM的预后判断及随访尚需更多前瞻性研究证实。

共识2：FCM检测CPCs具有预测SMM疾病进展的能力，CPCs水平升高是SMM的独立预后因素；建议在SMM诊断时通过FCM检测CPCs，并与sFLCR和M蛋白等常规检测相结合，以更好识别高危患者。推荐强度：建议采用。

3. MM：多项MM相关CPCs研究显示，在新诊断MM（NDMM）、auto-HSCT前后，CPCs高负荷预示着患者难以取得深度缓解，以及更短的无进展生存（PFS）期和总生存（OS）期，是MM患者独立的不良预后因素。因此将CPCs检测纳入MM的不同治疗阶段，可进一步动态评价患者预后。

NDMM初诊时检测CPCs在国外已成为常规检测[2]，可用于预判预后，完善R-ISS风险分层模型。已有多项国内外临床研究显示CPCs与MM预后显著相关（表1）。各中心在不同实验室条件下通过统计分析得出各自的cut-off值。FCM检测CPCs判断NDMM风险分级及预后时，采用欧洲流式学会（EuroFlow）二代流式细胞术（next generation flow，NGF）标准检测的cut-off值为≥0.010％，使用其他抗体组合方案检测的cut-off值有的低至≥0.002％、有的高至>0.070％（表1）。CPCs的cut-off值的差异可能与检测的细胞数目、用于检测CPCs的FCM的敏感性、治疗方案等因素有关。

**表1:** 各项研究中外周血循环浆细胞的临界值与结论

文献来源	疾病	设备	方法	样本量	检出率（%）^a^	cut-off值（%）^b^	阳性率（%）^c^	结论
Thorsteinsdóttir等[6]	NDMM/SMM/MGUS	ND	NGF	225	SMM：69.0；MM：93.0	≥0.002	SMM：22.0；MM：66.0	FCM检测CPCs有效鉴别MGUS、SMM和MM，帮助判断疾病进展
蒋元强等[11]	NDMM/RRMM	BCEpics XL	4色FCM	55	ND	≥0.010	ND	CPCs反映肿瘤负荷，预示疾病进展
Bae等[12]	NDMM	BD FACSCantoⅡ	5色FCM	85	74.1	≥0.020	67.1	CPCs≥0.020%与患者OS显著负相关
Gonsalves等[13]	NDMM	BD FACSCantoⅡ	6色FCM	566	67.0	CPCs：≥5/µl	26.0	结合CPCs定量可以显著提高R-ISS识别预后更差患者的能力，优化了R-ISS Ⅰ期和R-ISS Ⅱ期NDMM患者的危险分层
Cheng等[14]	NDMM	BD FACSCantoⅡ	7色FCM	191	59.2	>0.038	37.7	可以很好地区分肿瘤负担较重和反应率较低的患者（*P*<0.05），并可作为PFS和OS的独立预测指标
蔡梦洁等[15]	NDMM	BC Navios流式细胞仪	10色FCM	75	70.6	≥0.002	70.6	CPCs反映肿瘤负荷
Tembhare等[16]	NDMM	ND	10～13色高通量FCM	141	76.6	≥0.010	ND	MM患者治疗前和（或）治疗后进行FCM检测外周血CPCs，可以用于预测患者的预后
王中良等[17]	NDMM	BD FACSCantoⅡ	ND	72	55.6	≥0.010	55.6	CPCs反映病情，可预测现有危险分期
Garcés等[18]	NDMM	BD FACSCantoⅡ	EuroFlow™ NGF标准	374	92.0	≥0.010（0.06/µl）	ND	FCM检测CPCs可以提高R-ISS的分层能力
Bertamini等[19]	NDMM	ND	双管单平台FCM	401	67.0	>0.070	32.0	CPCs是独立的预后不良因素，纳入R-ISS模型后能改善R-ISS分层能力
Jelinek等[20]	NDMM	BD FACSCantoⅡ	EuroFlow™ NGF标准	590	不可移植：75.0 可移植：72.0	>2.000	不可移植：4.0 可移植：7.0	外周血CPCs>2.000%的患者可被视为一种超高风险的NDMM亚组对待，此类患者疾病特征高度类似于原发性PCL
Kostopoulos等[21]	NDMM	ND	NGF	525	89.1	≥0.010	ND	NDMM患者外周血高水平CPCs提示肿瘤有独特的生物学特征
Xia等[22]	NDMM	ND	8色FCM	301	ND	≥0.105	30.2	CPCs阳性患者应答及生存显著差于CPCs阴性患者；能进一步区分R-ISS Ⅱ期预后不良患者

**注** ^a^外周血检测到CPCs的患者比例。^b^文献中cut-off值的表达有3种方式：例1，10/50 000表示每50 000个外周血细胞检出10个异常克隆浆细胞；例2，0.01％表示异常克隆浆细胞在外周血细胞中的百分比；例3，0.06/µl表示每微升外周血检出0.06个异常克隆浆细胞。^c^外周血检测到CPCs≥cut-off值的患者比例。NDMM：新诊断多发性骨髓瘤；RRMM：复发难治多发性骨髓瘤；SMM：冒烟型骨髓瘤；MGUS：意义未明的单克隆免疫球蛋白血症；ND：无数据；BC：贝克曼库尔特；BD：碧迪医疗；EuroFlow™：欧洲流式学会；NGF：二代流式细胞术；FCM：流式细胞术；CPCs：循环浆细胞；OS：总生存；PFS：无进展生存；R-ISS：修订的国际分期系统；PCL：浆细胞白血病

CPCs能显著优化R-ISS风险模型的分层效果，在R-ISS中整合CPCs可显著提高识别高危患者的效率[13],[23]（2b）。一项梅奥研究将R-ISS分期为Ⅰ期和Ⅱ期且CPCs阳性（≥5/µl，相当于400/150 000）的患者定义为Ⅱb期，结果显示Ⅱb期患者的至下次治疗时间（time to next treatment，TTNT）和OS期明显短于Ⅰ期和Ⅱ期患者[13]（2b）。另外，CPCs结合PET-CT等方法，也能提高风险分层能力[14],[24]–[26]（2b）。有研究显示PET-CT高代谢病变部位>3个、CPCs≥0.01％是独立预后因素，两者联合能有效预测MM患者预后，与R-ISS分期结合能够进一步区分Ⅱ期及Ⅲ期PFS较差患者[24]。

共识3：CPCs阳性为NDMM独立预后不良因素；建议NDMM患者在初诊时通过FCM检测CPCs。推荐强度：强烈建议。

治疗后的CPCs状态与患者的预后显著相关。一项研究应用了外周血微小残留病（PBMRD）概念，在诊断时、3个疗程后（PBMRD1）和6个疗程后（PBMRD2），使用10～13色高敏感度多参数FCM检测CPCs（灵敏度10^−6^），PBMRD阳性定义为CPCs≥0.0001％。结果显示PBMRD1与PBMRD2中任一时间点的CPCs状态都与无事件生存（event-free survival，EFS）期和OS期显著相关[16]（2b）。

共识4：MM化疗后CPCs阳性预示缓解深度不足，并与更短的EFS和OS期相关，建议MM化疗后通过FCM检测CPCs，有助于识别预后不佳患者。推荐强度：建议采用。

auto-HSCT前后的CPCs状态与患者预后相关[2]。auto-HSCT造血干细胞采集前、移植前以及移植后100 d CPCs阳性患者的预后更差，PFS期、OS期和TTP明显变短[27]–[29]（2b）。auto-HSCT前后使用6色多参数FCM（灵敏度10^−4^），CPCs持续阴性患者获得严格意义的完全缓解（stringent complete response，sCR）的比例及生存情况均优于持续阳性、由阳转阴及由阴转阳患者[29]（2b）。

共识5：auto-HSCT前后CPCs状态是影响MM患者PFS期、OS期和TTP的独立预后因素。建议auto-HSCT前及移植后100 d进行CPCs FCM检测。推荐强度：建议采用。

4. 浆细胞白血病（PCL）：原发性PCL的侵袭性强，预后差，其诊断标准也在不断变化。2021年IMWG提出将外周血涂片浆细胞比例≥20％下降到≥5％作为PCL的诊断标准[30]。而最新研究提出根据FCM检测结果将比例进一步下降到2％[20]（2b）。

Jelinek等[20]研究表明，EuroFlow NGF检测CPCs在2％～5％的MM患者和CPCs在5％～20％的MM患者中位PFS期相近（3.4个月对5.1个月，*P*＝0.42），且PFS期都明显短于CPCs<2％的MM患者（2b）。

共识6：CPCs≥5％是诊断PCL的标准，EuroFlow NGF检测CPCs≥2％的患者可能具有PCL特征，建议初诊怀疑PCL时通过FCM检测CPCs，识别具有潜在PCL特征的超高危MM患者。推荐强度：建议采用。

二、FCM检测CPCs的技术要点

FCM检测CPCs的技术包括常规的多参数FCM和EuroFlow为代表的NGF技术，后者显示出更高的方法学灵敏度。EuroFlow NGF方案使用双管8色抗体组合[31]，共收集1×10^7^个细胞，最低检出限（limit of detection，LOD）可达3×10^−6^［30/（1×10^7^）］，最低定量限（lower limit of quantification，LLOQ）可达5×10^−6^［50/（1×10^7^）］。此外，该方案还建立了标准化的检测分析流程，有利于促进多中心临床试验的结果同质化。但该方案需要更多样本（5～10 ml）、成本高、数据量大、分析效率低，目前在我国尚难普遍应用。Sato等[32]对治疗后MM患者采用单管10色方案监测微小残留病（MRD），获取5.5×10^6^个细胞，LLOQ达到1×10^−5^，与EuroFlow NGF方案对比，该方案节约样本的同时，成本也可降低约60％，并且与NGF有较高的结果一致性。结合国内检测现状，常规多参数FCM检测CPCs更具有普适性，本共识重点对常规多参数FCM检测CPCs的技术要点进行推荐。

1. 抗体选择：CPCs的免疫表型与骨髓中克隆性浆细胞的免疫表型相似。典型的正常浆细胞为CD38^bri^ CD138^+^CD19^+^CD56^−^CD45^+^CD27^bri^ CD81^+^CD200^−^CD28^−^CD117^−^CD20^−^，克隆性浆细胞可出现许多非典型免疫表型如CD19阴性，CD138表达增强，CD38、CD45、CD27、CD81表达减弱以及异常表达CD56、CD200、CD28、CD117、CD20等，并且这些表型常常共同出现在同一群克隆性浆细胞中。值得注意的是，正常浆细胞中部分亚群也可存在非典型免疫表型，但通常不会同时出现多种非典型免疫表型。因此在CD38、CD138、CD45圈定总浆细胞及CD19、CD56初步区分正常和异常浆细胞的基础上，建议联用CD27、CD81、CD117等更多标志以提高对克隆性浆细胞的识别能力，其中CD27在克隆性浆细胞中发生异常的频率较高，可优先包含在检测组合中[33]。检测胞质免疫球蛋白轻链（cyκ和cyλ）的限制性是确定浆细胞克隆性最直观的证据，但因洗涤、破膜的步骤繁琐易造成细胞丢失，国际上对于是否检测cyκ和cyλ仍具争议。陈文明教授团队用骨髓标本对比了只含胞膜抗原和包含cyκ/cyλ的两种单管8色方案对MM MRD的检测能力（LOD为6×10^−6^），结果显示包含cyκ/cyλ的单管方案识别肿瘤性浆细胞的准确性更高，并且两种检测方案的总浆细胞比例差异无统计学意义，提示破膜对浆细胞比例影响较小[34]。结合上述经验，我们建议将cyκ和cyλ纳入检测方案中。综上，CPCs检测方案建议优先采用CD38、CD138、CD45、CD19、CD56、CD27、cyκ和cyλ组成的单管8色检测方案[35]，有条件实验室可采用双管8色或单管10色方案，通过增加检测抗体种类如CD117、CD81等进一步提高检测敏感性。抗体克隆号和荧光偶联的选择对于组合方案的检测性能也十分关键，各实验室可根据流式细胞仪参数配置进行抗体组合搭配，可参考《多参数流式细胞术检测急性白血病及浆细胞肿瘤微小残留病中国专家共识》[35]及EuroFlow抗体组合方案[31]。

靶向CD38的单克隆抗体如达雷妥尤单抗（Daratumumab）和伊莎妥昔单抗（Isatuximab）已被批准用于治疗MM，但此类单抗药物会干扰浆细胞表面CD38的检测，导致假阴性结果[36]。因此，建议应用此类药物的患者检测CPCs时使用多表位CD38 Me[31]，或使用可识别内质网膜上的CLIMP-63蛋白的VS38c以及CD38纳米单抗等代替[37]–[38]。其他抗体如CD319、CD229、CD54等在浆细胞中高表达，与CD45、CD138等组合作为单抗治疗后的浆细胞的圈门抗体仍有待更多研究证实[39]–[40]。

2. 检测灵敏度选择：我们建议以30个和50个细胞分别作为CPCs的方法学检测限和可定量的最小细胞数，则LOD＝30/获取细胞数×100％，LLOQ＝50/获取细胞数×100％。Sanoja-Flores等[41]汇总了多项研究，综述了LOD在10^−4^情况下，CPCs在新诊断MGUS、SMM、MM及治疗后MM患者中的阳性率分别为25％、24％、67％、19％；采用NGF使LOD达到10^−6^时，CPCs阳性率可分别提高至59％、100％、100％、26％，提示方法学灵敏度越高，可能临床意义更大。结合前述多项研究，对新诊断SMM和MM患者，CPCs检测的LOD达到10^−4^即可辅助预后判断，且该检测灵敏度对所需标本量、分析软件等要求不高，更易推广和实施，因此建议CPCs检测的LOD至少达到10^−4^，有条件实验室可增加获取细胞数，使LOD或LLOQ达到10^−5^或更低。对MGUS患者及治疗后MM患者的CPCs监测，LOD或LLOQ达到10^−5^或更低可提供更好的研究价值，有条件实验室可采用。

共识7：建议FCM检测CPCs的LOD至少达到10^−4^，检测方案建议包含胞质轻链的抗体组合方案，推荐CD38、CD138、CD45、CD19、CD56、CD27、cyκ和cyλ组成的单管8色检测方案，有条件实验室可采用双管8色或单管10色方案。采用EuroFlow NGF方案的实验室采用NGF标准下的适当cut-off值，未达EuroFlow NGF标准的实验室可通过各自标准化的FCM操作流程、合理的抗体组合方案设计以及严谨的临床研究分析，建立各自中心的cut-off值。推荐强度：建议采用。

3. 样本储存及制备：由于使用肝素抗凝时，CD138的表达会减弱，建议采用EDTA抗凝。随着标本放置时间延长，容易出现CD138抗原脱落，建议在24 h内完成检测，尽量不超过48 h；如样本放置超过48 h，应注意存在假阴性可能，须在报告中注明。

标本染色前，需先对外周血计数以决定用血体积。对于治疗后MM患者，有条件实验室可获取（2～3）×10^6^个细胞/管，使LOD达到10^−5^，可对足量外周血进行富集，通过氯化铵溶红的步骤，将细胞总体积控制在200 µl，再进行抗体标记。为LLOQ达到10^−5^，则需至少获取5×10^6^个细胞/管，建议参考EuroFlow NGF的标准标本制备流程。不建议采用Ficoll密度梯度离心法富集单个核细胞，因为Ficoll富集过程会造成浆细胞的选择性损失，并加速CD138等抗原丢失[42]–[43]。

获取（2～3）×10^6^个白细胞的细胞富集及染色流程如下：①外周血计数，吸取足量外周血至15 ml离心管中，继续加满PBS溶液，37 °C温水浴5 min，800×*g*离心5 min，吸去上清；②加入红细胞裂解液（NH_4_Cl）至15 ml，充分混匀，溶血6 min，800×*g*离心5 min，吸去上清；③加入PBS至4 ml（分2次），吹打混匀后全部转移至流式管中，540×*g*离心5 min，吸去上清；④加入胞膜抗体，混匀，避光孵育15 min；⑤加入A液（BD IntraSure™ Kit）150 µl，混匀，避光孵育6 min；⑥加入溶血素（BD FACS™ Lysing Solution）2 ml，避光溶血10 min，540×*g*离心5 min，吸去上清；⑦加入B液（BD IntraSure™ Kit）75 µl，加入Kappa和Lambda抗体，混匀，避光孵育15 min；⑧加入PBS 2 ml，混匀后540×*g*离心5 min，吸去上清；⑨加入500 µl PBS溶液上机检测。

4. 细胞获取、设门分析及报告要求：由于肿瘤浆细胞FSC参数变异较大，建议控制FSC电压避免肿瘤细胞群超出右界限，并设定合适的FSC阈值，以尽量减少细胞碎片的获取比例。应注意外周血正常浆细胞是B细胞的终末分化阶段，浆细胞呈现持续成熟、逐渐获得浆细胞相关标志CD138和CD38的特点，因此CD138呈现从无到有的异质性表达模式，且大部分为阴性，CD38则强度稍弱于骨髓中正常浆细胞[41]。建议圈总浆细胞门时，应圈入CD38^bri^CD138^−^细胞群，以防止漏掉正常浆细胞或CD138阴性的异常浆细胞。

建议以单克隆浆细胞占白细胞百分比的方式进行报告，该计算方法不受红细胞裂解效果和细胞碎片的影响。白细胞总数应包含CD45阳性区白细胞和CD45阴性区的异常浆细胞。建议报告LOD及LLOQ并参考国际临床细胞计量学学会（International Clinical Cytometry Society，ICCS）MM FCM MRD建议[44]：①检测结果>LLOQ：报告异常单克隆浆细胞百分比、异常单克隆浆细胞数、白细胞总数；②检测结果介于LOD～LLOQ之间：报告异常单克隆浆细胞百分比<LLOQ、异常单克隆浆细胞数、白细胞总数；③检测结果<LOD：报告异常单克隆浆细胞百分比<LOD、异常单克隆浆细胞数、白细胞总数。

设门分析策略以图1为例：利用CD45/Time选取液流稳定的区间（P1），然后通过FSC-H/FSC-A、SSC-H/SSC-A（P2-1、P2-2）去除黏连体，再通过SSC-A/FSC-A（P3）去除细胞碎片（图1A～D）；在P3中圈出CD38^+^CD138^+^及CD38^bri^CD138^−^细胞群（P4），然后通过CD45联合SSC在P4中圈出精确的浆细胞群（PC）（图1E、F）；根据CD56和CD19的表达情况将PC分群，该例可分为CD56^+^CD19^−^群（P5）和CD56^−^CD19^+^群（P6）（图1G）；P5细胞群的CD27和CD45表达均低于P6细胞群，且限制性表达cyKappa，判定为表型异常的单克隆浆细胞。P6为正常的多克隆浆细胞（图1H～J）；在P3中圈出淋巴细胞（lym），以lym中CD19阳性成熟B细胞（mature B）作为背景细胞，辅助确定cyKappa和cyLambda分界线（图1K、L、O）；利用FSC-H/FSC-A、SSC-A/FSC-A观察P5细胞群的集中程度（图1M、N）；在P3中圈CD45阳性细胞群（P7），参考P7门在PC中圈CD45阴性的浆细胞群（P8），以P7（2 016 481个）与P8（57个）数量之和作为白细胞数量（2 016 538个）（图1O、P）。该例报告结果：共获取2 016 538个白细胞，其中71个细胞（占总白细胞0.004％）表达CD38、CD138、cyKappa、CD56、CD27dim，不表达cyLambda、CD19、CD45，为表型异常的单克隆浆细胞；另可见97个细胞（占总白细胞0.005％）为正常浆细胞，LOD：0.0015％（30/2 016 538×100％），LLOQ：0.0025％（50/2 016 538×100％）。

**图1:**
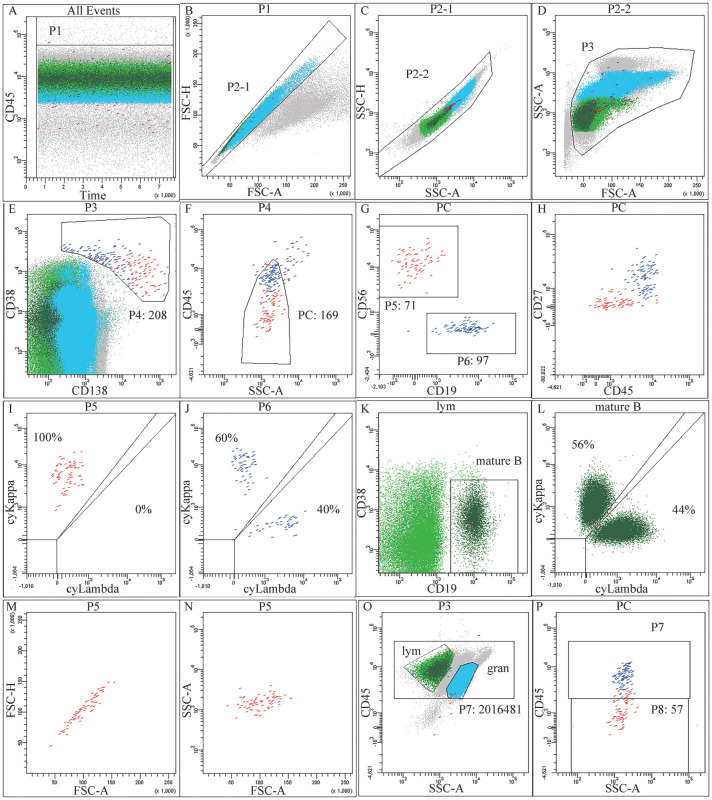
流式细胞术检测的典型散点图

三、FCM检测CPCs的未来展望

FCM检测CPCs的临床应用价值日益凸显，未来的发展涉及到检测方法标准化、抗体组合持续优化和cut-off值一致化等方面。cut-off值的统一虽然很重要，但因硬件、软件、成本、操作等多方面影响，在目前阶段难度较大。同一区域或相近地区的医疗机构形成局域性统一的cut-off值，或许是可行且有益的方法。

## References

[1] Rodriguez-Otero P, Paiva B, San-Miguel JF (2021). Roadmap to cure multiple myeloma[J]. Cancer Treat Rev.

[2] Marcon C, Simeon V, Deias P (2022). Experts' consensus on the definition and management of high risk multiple myeloma[J]. Front Oncol.

[3] Mouhieddine TH, Weeks LD, Ghobrial IM (2019). Monoclonal gammopathy of undetermined significance[J]. Blood.

[4] Han JH, Wang JN, Zhang YL (2020). Prevalence of monoclonal gammopathy of undetermined significance in a large population with annual medical check-ups in China[J]. Blood Cancer J.

[5] Sanoja-Flores L, Flores-Montero J, Garcés JJ (2018). Next generation flow for minimally-invasive blood characterization of MGUS and multiple myeloma at diagnosis based on circulating tumor plasma cells (CTPC)[J]. Blood Cancer J.

[6] Thorsteinsdóttir S, Oskarsson J Þ, Rögnvaldsson S (2022). Circulating Tumor Plasma Cells in the Screened Istopmm Smoldering Multiple Myeloma Cohort[J]. Blood.

[7] Thorsteinsdottir S, Kristinsson SY (2022). The consultant's guide to smoldering multiple myeloma[J]. Hematology Am Soc Hematol Educ Program.

[8] Kyle RA, Rajkumar SV (2007). Epidemiology of the plasma-cell disorders[J]. Best Pract Res Clin Haematol.

[9] Gonsalves WI, Rajkumar SV, Dispenzieri A (2017). Quantification of circulating clonal plasma cells via multiparametric flow cytometry identifies patients with smoldering multiple myeloma at high risk of progression[J]. Leukemia.

[10] Termini R, Žihala D, Terpos E (2022). Circulating Tumor and Immune Cells for Minimally Invasive Risk Stratification of Smoldering Multiple Myeloma[J]. Clin Cancer Res.

[11] 蒋 元强, 李 建勇, 吴 雨洁 (2006). 多发性骨髓瘤患者外周血与骨髓中瘤细胞的检测及临床意义[J]. 中国实验血液学杂志.

[12] Bae MH, Park CJ, Kim BH (2018). Increased circulating plasma cells detected by flow cytometry predicts poor prognosis in patients with plasma cell myeloma[J]. Cytometry B Clin Cytom.

[13] Gonsalves WI, Jevremovic D, Nandakumar B (2020). Enhancing the R-ISS classification of newly diagnosed multiple myeloma by quantifying circulating clonal plasma cells[J]. Am J Hematol.

[14] Cheng Q, Cai L, Zhang Y (2021). Circulating Plasma Cells as a Biomarker to Predict Newly Diagnosed Multiple Myeloma Prognosis: Developing Nomogram Prognostic Models[J]. Front Oncol.

[15] 蔡 梦洁, 梅 仁, 戴 兰 (2021). 多发性骨髓瘤循环肿瘤浆细胞的免疫表型特征及其临床意义[J]. 临床输血与检验.

[16] Tembhare PR, Sriram H, Khanka T (2022). Circulating Clonal Plasma Cells at Diagnosis and Peripheral Blood Measurable Residual Disease Assessment Provide Powerful Prognostication Biomarkers in Newly-Diagnosed Multiple Myeloma Patients Treated without Autologous Transplant[J]. Blood.

[17] 王 中良, 邢 鹏涛, 王 金慧 (2022). 多发性骨髓瘤中循环骨髓瘤细胞水平及临床意义[J]. 临床骨科杂志.

[18] Garcés JJ, Cedena MT, Puig N (2022). Circulating Tumor Cells for the Staging of Patients with Newly Diagnosed Transplant-Eligible Multiple Myeloma[J]. J Clin Oncol.

[19] Bertamini L, Oliva S, Rota-Scalabrini D (2022). High Levels of Circulating Tumor Plasma Cells as a Key Hallmark of Aggressive Disease in Transplant-Eligible Patients with Newly Diagnosed Multiple Myeloma[J]. J Clin Oncol.

[20] Jelinek T, Bezdekova R, Zihala D (2023). More Than 2％ of Circulating Tumor Plasma Cells Defines Plasma Cell Leukemia-Like Multiple Myeloma[J]. J Clin Oncol.

[21] Kostopoulos IV, Ntanasis-Stathopoulos I, Rousakis P (2023). Circulating Plasma Cells in Newly Diagnosed Multiple Myeloma: Prognostic and More[J]. J Clin Oncol.

[22] Xia Y, Zhang R, Jin Y (2022). Clinical Significance and Molecular Characteristics of Circulating Plasma Cells in Multiple Myeloma [J]. Blood.

[23] Galieni P, Travaglini F, Vagnoni D (2021). The detection of circulating plasma cells may improve the Revised International Staging System (R-ISS) risk stratification of patients with newly diagnosed multiple myeloma[J]. Br J Haematol.

[24] Abe Y, Narita K, Kobayashi H (2019). Pretreatment (18)F-FDG PET/CT combined with quantification of clonal circulating plasma cells as a potential risk model in patients with newly diagnosed multiple myeloma[J]. Eur J Nucl Med Mol Imaging.

[25] Papadhimitriou SI, Terpos E, Liapis K (2022). The Cytogenetic Profile of Primary and Secondary Plasma Cell Leukemia: Etiopathogenetic Perspectives, Prognostic Impact and Clinical Relevance to Newly Diagnosed Multiple Myeloma with Differential Circulating Clonal Plasma Cells[J]. Biomedicines.

[26] Hofste op Bruinink D, Kuiper R, van Duin M (2022). Identification of High-Risk Multiple Myeloma With a Plasma Cell Leukemia-Like Transcriptomic Profile[J]. J Clin Oncol.

[27] Dingli D, Nowakowski GS, Dispenzieri A (2006). Flow cytometric detection of circulating myeloma cells before transplantation in patients with multiple myeloma: a simple risk stratification system[J]. Blood.

[28] Chakraborty R, Muchtar E, Kumar SK (2016). Risk stratification in myeloma by detection of circulating plasma cells prior to autologous stem cell transplantation in the novel agent era[J]. Blood Cancer J.

[29] Chakraborty R, Muchtar E, Kumar SK (2017). Serial measurements of circulating plasma cells before and after induction therapy have an independent prognostic impact in patients with multiple myeloma undergoing upfront autologous transplantation[J]. Haematologica.

[30] Fernández de Larrea C, Kyle R, Rosiñol L (2021). Primary plasma cell leukemia: consensus definition by the International Myeloma Working Group according to peripheral blood plasma cell percentage[J]. Blood Cancer J.

[31] Flores-Montero J, Sanoja-Flores L, Paiva B (2017). Next Generation Flow for highly sensitive and standardized detection of minimal residual disease in multiple myeloma[J]. Leukemia.

[32] Sato K, Okazuka K, Ishida T (2021). Minimal residual disease detection in multiple myeloma: comparison between BML single-tube 10-color multiparameter flow cytometry and EuroFlow multiparameter flow cytometry[J]. Ann Hematol.

[33] Cannizzo E, Carulli G, Del Vecchio L (2012). The role of CD19 and CD27 in the diagnosis of multiple myeloma by flow cytometry: a new statistical model[J]. Am J Clin Pathol.

[34] Wang XF, Wang TT, Zhang ZY (2019). Comparison of minimal residual disease in multiple myeloma patients detected by 8-color panels and next generation flow cytometry[J]. Chin J Hematol.

[35] 中国免疫学会血液免疫分会临床流式细胞术学组 (2017). 多参数流式细胞术检测急性白血病及浆细胞肿瘤微小残留病中国专家共识(2017年版)[J]. 中华血液学杂志.

[36] Oberle A, Brandt A, Alawi M (2017). Long-term CD38 saturation by daratumumab interferes with diagnostic myeloma cell detection[J]. Haematologica.

[37] Mizuta S, Kawata T, Kawabata H (2019). VS38 as a promising CD38 substitute antibody for flow cytometric detection of plasma cells in the daratumumab era[J]. Int J Hematol.

[38] Pape LJ, Hambach J, Gebhardt AJ (2022). CD38-specific nanobodies allow in vivo imaging of multiple myeloma under daratumumab therapy[J]. Front Immunol.

[39] Tembhare PR, Ghogale S, Tauro W (2018). Evaluation of CD229 as a new alternative plasma cell gating marker in the flow cytometric immunophenotyping of monoclonal gammopathies[J]. Cytometry B Clin Cytom.

[40] Soh KT, Tario JD, Hahn T (2021). CD319 (SLAMF7) an alternative marker for detecting plasma cells in the presence of daratumumab or elotuzumab[J]. Cytometry B Clin Cytom.

[41] Sanoja-Flores L, Flores-Montero J, Pérez-Andrés M (2020). Detection of Circulating Tumor Plasma Cells in Monoclonal Gammopathies: Methods, Pathogenic Role, and Clinical Implications[J]. Cancers (Basel).

[42] 中国免疫学会血液免疫分会临床流式细胞术学组 (2019). 白血病/淋巴瘤免疫分型检测质量控制指南[J]. 检验医学.

[43] Soh KT, Wallace PK (2021). Evaluation of measurable residual disease in multiple myeloma by multiparametric flow cytometry: Current paradigm, guidelines, and future applications[J]. Int J Lab Hematol.

[44] Arroz M, Came N, Lin P (2016). Consensus guidelines on plasma cell myeloma minimal residual disease analysis and reporting[J]. Cytometry B Clin Cytom.

